# LncRNA H19 diminishes dopaminergic neuron loss by mediating microRNA-301b-3p in Parkinson’s disease via the HPRT1-mediated Wnt/β-catenin signaling pathway

**DOI:** 10.18632/aging.102877

**Published:** 2020-05-20

**Authors:** Jingjing Jiang, Xuanyu Piao, Siying Hu, Jingbo Gao, Min Bao

**Affiliations:** 1 Department of Anesthesiology, Shengjing Hospital of China Medical University, Shenyang 110004, P.R. China; 2 Department of Neurosurgery, Shengjing Hospital of China Medical University, Shenyang 110004, P.R. China

**Keywords:** long noncoding RNA H19, microRNA-301b-3p, hypoxanthine phosphoribosyltransferase 1, Parkinson’s disease, dopaminergic neuron

## Abstract

Long non-coding RNAs (lncRNA) and microRNAs (miRNAs) are a subject of active investigation in neurodegenerative disorders including Parkinson’s disease (PD). We hypothesized a regulatory role of lncRNA H19 with involvement of hypoxanthine phosphoribosyltransferase 1 (HPRT1) in dopaminergic neuron loss in PD model mice obtained by 6-hydroxydopamine (6-OHDA) lesions. We predicted the differentially expressed genes and related mechanisms by microarray analysis. We measured the expression of tyrosine hydroxylase (TH) and proneural genes in the substantia nigra of lesioned mice before and after treatment with lentiviral oe-HPRT1, agomir-miR-301b-3p and inhibition of the Wnt/β-catenin pathway. We also evaluated the relationship among lncRNA H19, HPRT1 and miR-301b-3p as well as the Wnt/β-catenin signaling pathway in these mice. The obtained results predicted and further confirmed a low level of HPRT1 in lesioned mice. We found low expression of lncRNA H19 and showed that its forced overexpression regulated HPRT1 by binding to miR-301b-3p. The overexpression of HPRT1 increased TH expression and inhibited dopaminergic neuron loss *via* activating the Wnt/β-catenin pathway, as reflected by increased expressions of Nurr-1, Pitx-3, Ngn-2 and NeuroD1. Thus, overexpressed lncRNA H19 protects against dopaminergic neuron loss in this PD model through activating the Wnt/β-catenin pathway *via* impairing miR-301b-3p-targeted inhibition of HPRT1 expression.

## INTRODUCTION

Parkinson’s disease (PD) is reported to be the commonest neurodegenerative disorder, plaguing approximately 1% of those aged above 65 years across the globe [1]. The pathological characteristics of PD include dopaminergic neuron loss in the substantia nigra as well as the accumulation of Lewy bodies composed of synaptic protein α-synuclein [2, 3]. Patients suffering from PD are greatly vulnerable to cognitive decline notably including executive deficits beginning at an early phase of the disease [4]. In addition to typical motor symptoms, PD patients also manifest such neuropsychiatric symptoms as anxiety, depression and motivational deficits [5]. Although the dopamine replacement therapies can typically the alleviate motor symptoms, their efficacy declines with PD progression [6]. Neuroprotective therapies addressing non-motor and non-dopaminergic aspects without provoking serious side effects are actively investigated [7]. Therefore, studies are required to better understand the molecular events of PD, and characterize the potential biomarkers and pathways for treatment [8].

Hypoxanthine-guanine phosphoribosyltransferase (HPRT) is a cytoplasmic enzyme widely distributed in the body [9], and the hypoxanthine phosphoribosyltransferase 1 (HPRT1) gene is on the long arm of the X chromosome (Xq26.1) [10]. HPRT deficiency impairs functions of the dopaminergic neurons and dopamine pathways, such as accelerated axonal and neuronal degeneration and aberrant nigrostriatal axonal arborization [11, 12]. Specifically, the neuro-regulatory defects attributed to HPRT deficiency are involved in dysregulation of the Wnt/ β-catenin signaling pathway, in addition to reduced expression of dopaminergic transcription factors [13]. As shown by L’Episcopo et al. [14], the Wnt/β-catenin signaling pathway has a pivotal role in the neurogenesis of dopaminergic neurons in the midbrain. Interestingly, the activation of the Wnt/β-catenin signaling pathway could be stimulated by treatment with long noncoding RNA (lncRNA) H19, which had controlling effects on cellular metabolism [15, 16]. In recent years, the participation of non-coding RNAs, especially lncRNAs and microRNAs (miRNAs) has been extensively documented in multiple studies in neurodegenerative disorders including PD [17, 18]. Other studies show that H19 is highly expressed in various malignant tumors, where it serves as an oncogene [19, 20]. Results of studies *in silico* predict that H19 and HPRT1 both directly bind to microRNA-301b-3p (miR-301b-3p). Alvarez-Erviti et al. suggested that the expression of miR-301b was significantly increased in the *substantia nigra pars compacta* in PD [21]. Accordingly, the present study was performed to test our hypothesis that the dopaminergic neuron loss in PD could be regulated by lncRNA H19 by a mechanism implicated in the HPRT1-dependent Wnt/β-catenin signaling pathway and miR-301b-3p.

## RESULTS

### HPRT1 is poorly expressed in brain tissues of mice in 6-OHDA-induced PD mouse model

**Figure 1:**
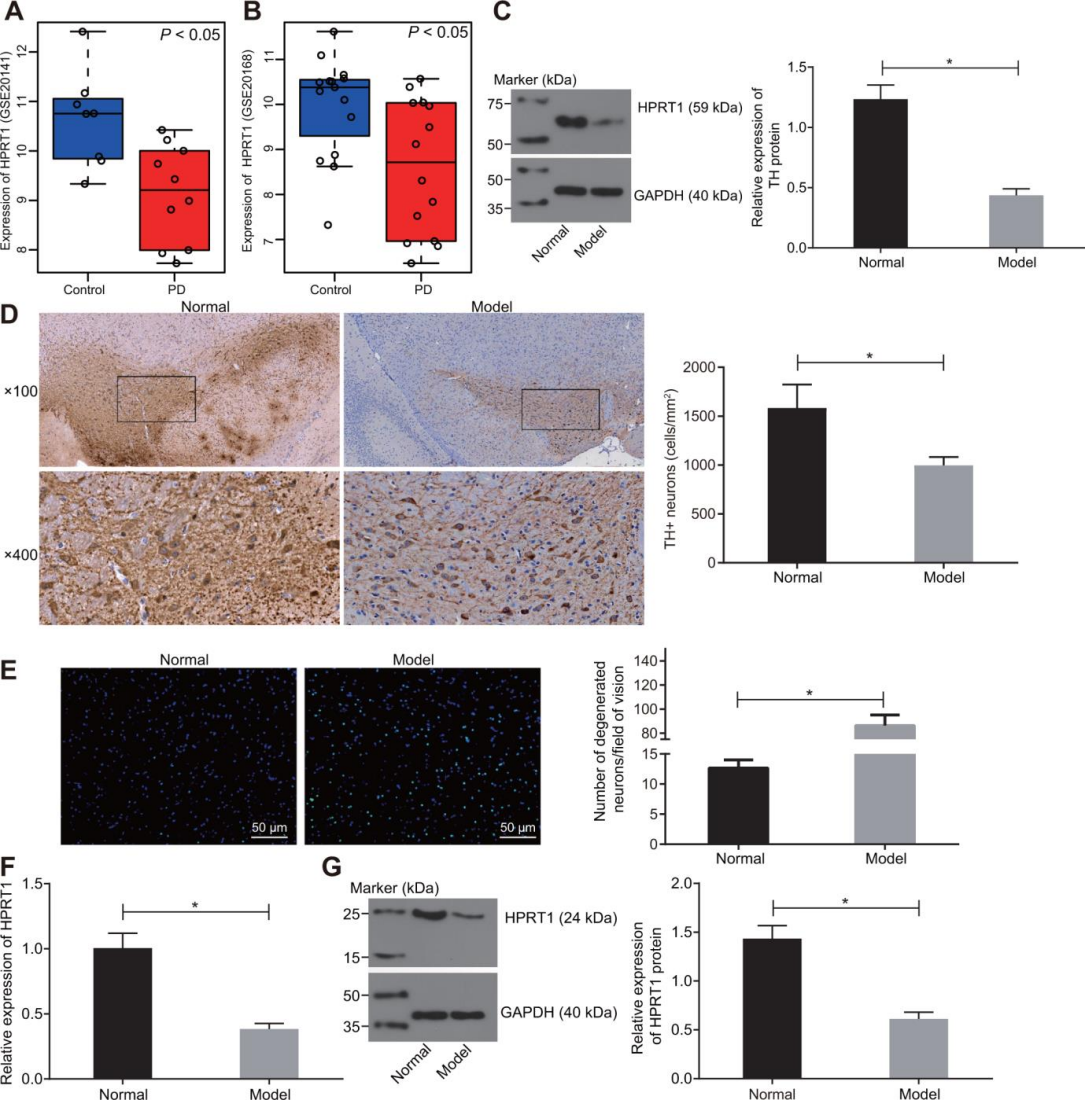
**HPRT1 is poorly expressed in the substantia nigra tissues of 6-OHDA-induced PD mice.** (**A**) The expression of HPRT1 in the expression profile of GSE20141 related to PD; (**B**) The expression of HPRT1 in the expression profile of GSE20168 related to PD. (**C**) The protein expression of TH in the substantia nigra tissues of 6-OHDA-induced PD mice measured by western blot analysis. (**D**) Immunohistochemical analysis for the TH positive cells in the substantia nigra tissues of 6-OHDA-induced PD mice (upper × 100, lower × 400); (**E**) Fluoro-Jade B-stained apoptotic neurons (scale bar = 50 μm). (**F**) mRNA expression of HPRT1 in the substantia nigra tissues examined by RT-qPCR; (**G**) Protein expression of HPRT1 in the substantia nigra tissues examined by western blot assay. *p < 0.05. n = 6. Measurement data are by means ± standard deviation. Comparison between two groups was analyzed by independent sample *t* test.

Expression profiles of GSE20141 and GSE20168 were retrieved from the GEO database. The differential expression analysis on the healthy control samples and PD samples in the profile indicated that HPRT1 was poorly expressed in PD (Figure 1A, 1B). In our study, 6-OHDA-induced dopaminergic neuron injury in mice was used as an animal model of PD. Furthermore, substantia nigra was extracted from our PD mice to determine expression of TH, a key enzyme in the dopamine synthesis pathway, by western blot assay [22]. Relative to control mice, there was low residual TH expression in the substantia nigra tissues of 6-OHDA-induced PD mice (Figure 1C). At the same time, the immunohistochemical analysis revealed a significant reduction in the number of TH-positive dopamine neurons in the lesioned substantia nigra tissues of the 6-OHDA-induced PD mice was lower than that in control mice (Figure 1D). These indicated that injection of 6-OHDA led to nigrostriatal dopamine degeneration. The data from Fluoro-Jade B staining showed that 6-OHDA infusion increased the apoptosis rate of neurons (Figure 1E). The HPRT1 expression was lower in the substantia nigra tissues of 6-OHDA-induced PD mice, as examined by RT-qPCR and Western blot assay (Figure 1F, 1G). Overall, the results indicate that HPRT1 may be a key player in 6-OHDA -mediated dopamine loss.

### Overexpressed HPRT1 inhibits dopaminergic neuron loss in 6-OHDA-induced PD mice

**Figure 2:**
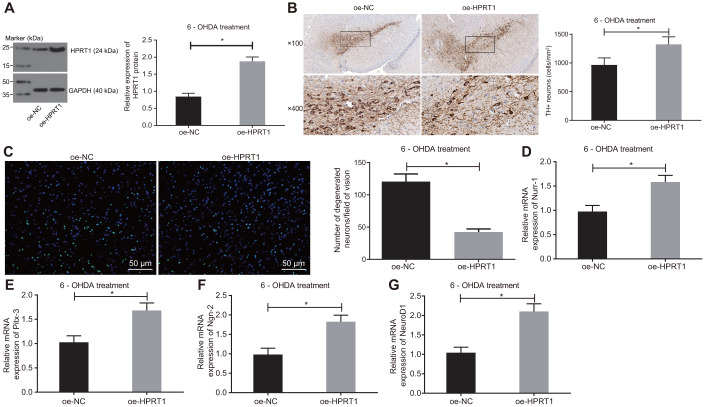
**Overexpressed HPRT1 inhibits dopaminergic neuron loss in 6-OHDA-induced PD mice.** 6-OHDA-induced PD mice were treated with oe-NC or oe-HPRT1. (**A**) Protein expression of HPRT1 in the substantia nigra tissues examined by western blot assay. (**B**) TH positive neurons in the substantia nigra examined by immunohistochemistry (upper × 100, lower × 400). (**C**) Fluoro-Jade B-stained apoptotic neurons (scale bar = 50 μm). (**D**–**G**) The mRNA expression of Nurr-1 (**D**), Pitx-3 (**E**), Ngn-2 (**F**) and NeuroD1 (**G**) in the substantia nigra tissues examined by RT-qPCR. *p < 0.05. n = 6. Measurement data are by means ± standard deviation. Comparison between two groups was analyzed by independent sample *t* test.

To further examine the effect of HPRT1 on the dopaminergic neuron loss in 6-OHDA-induced PD mice, we injected lentiviral oe-NC or oe-HPRT1 into the 6-OHDA-induced PD mice, followed by detection of protein expression of HPRT1 in the substantia nigra tissues by western blot assay. The PD model mice co-injected with lentiviral oe-HPRT1 displayed significantly increased HPRT1 expression (Figure 2A). Next, we determined the number of immunohistochemically TH-positive neurons in the substantia nigra, results of which suggested rescue of dopamine neurons in PD mice co-injected with lentiviral oe-HPRT1 (Figure 2B). The data from Fluoro-Jade B staining exhibited that the apoptosis rate of neurons was reduced after injection of lentiviral oe-HPRT1 (Figure 2C). Then, RT-qPCR was used to measure mRNA expression of such proneural genes as Nurr-1 (Figure 2D), Pitx-3 (Figure 2E), Ngn-2 (Figure 2F) and NeuroD1 (Figure 2G), which proved to be significantly increased after co-injection of lentiviral oe-HPRT1 along with 6-OHDA. These results indicated that HPRT1 attenuated the dopaminergic neuron loss in 6-OHDA-induced PD mice.

### Overexpressed HPRT1 activates the Wnt/β-catenin signaling pathway in N27 dopaminergic neurons

**Figure 3:**
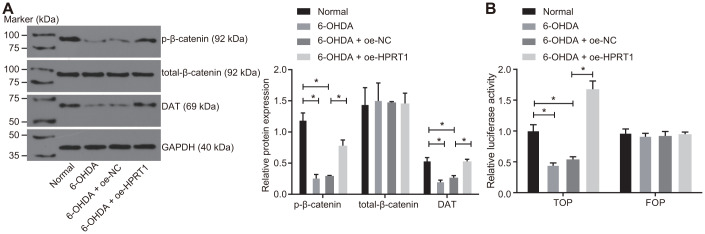
**Overexpressed HPRT1 activates the Wnt/β-catenin signaling pathway in a PD model.** 6-OHDA-treated N27 dopaminergic neurons were treated with oe-NC or oe-HPRT1. (**A**) The protein expression of total-β-catenin and DAT as well as the extent of β-catenin phosphorylation in the N27 dopaminergic neurons detected by western blot assay. (**B**) The activity of the Wnt/β-catenin signaling pathway expressed by TOP/FOP ratio using TOP/FOP flash reporter assay. FOP was designated as background value or as negative control due to its stability. *p < 0.05. Measurement data from three independent experiments are expressed by means ± standard deviation. Comparison between two groups was analyzed by unpaired *t* test.

Previous studies have proved that depletion of HPRT1 or β-catenin suppresses the development of dopaminergic neurons [23, 24]. Moreover, the depletion of HPRT1 expression could repress the activation of the Wnt/β-catenin [13]. We hypothesized that the effects of HPRT1 exerted in the PD model were achieved through the Wnt/β-catenin signaling pathway. Following this, we measured the levels of total-β-catenin and the extent of β-catenin phosphorylation in the N27 dopaminergic neurons by western blot assay, finding that N27 dopaminergic neurons treated with 6-OHDA showed a significantly reduced extent of β-catenin phosphorylation and DAT expression, while overexpression of HPRT1 could rescue the declines in the extent of β-catenin phosphorylation and DAT expression induced by 6-OHDA (Figure 3A). The TOP/FOP flash assay also showed decreased TOP flash luciferase activity in the N27 dopaminergic neurons treated with 6-OHDA, suggesting the Wnt/β-catenin signaling pathway was blocked in N27 dopaminergic neurons. Following oe-HPRT1 treatment in the presence of 6-OHDA, N27 dopaminergic neurons demonstrated increased TOP flash luciferase activity (Figure 3B), as revealed by the TOP/FOP flash assay. These findings verified the activation of the Wnt/β-catenin signaling pathway was stimulated by overexpression of HPRT1.

### Overexpressed HPRT1 inhibits dopaminergic neuron loss via the Wnt/β-catenin signaling pathway in mice in 6-OHDA-induced PD model

**Figure 4:**
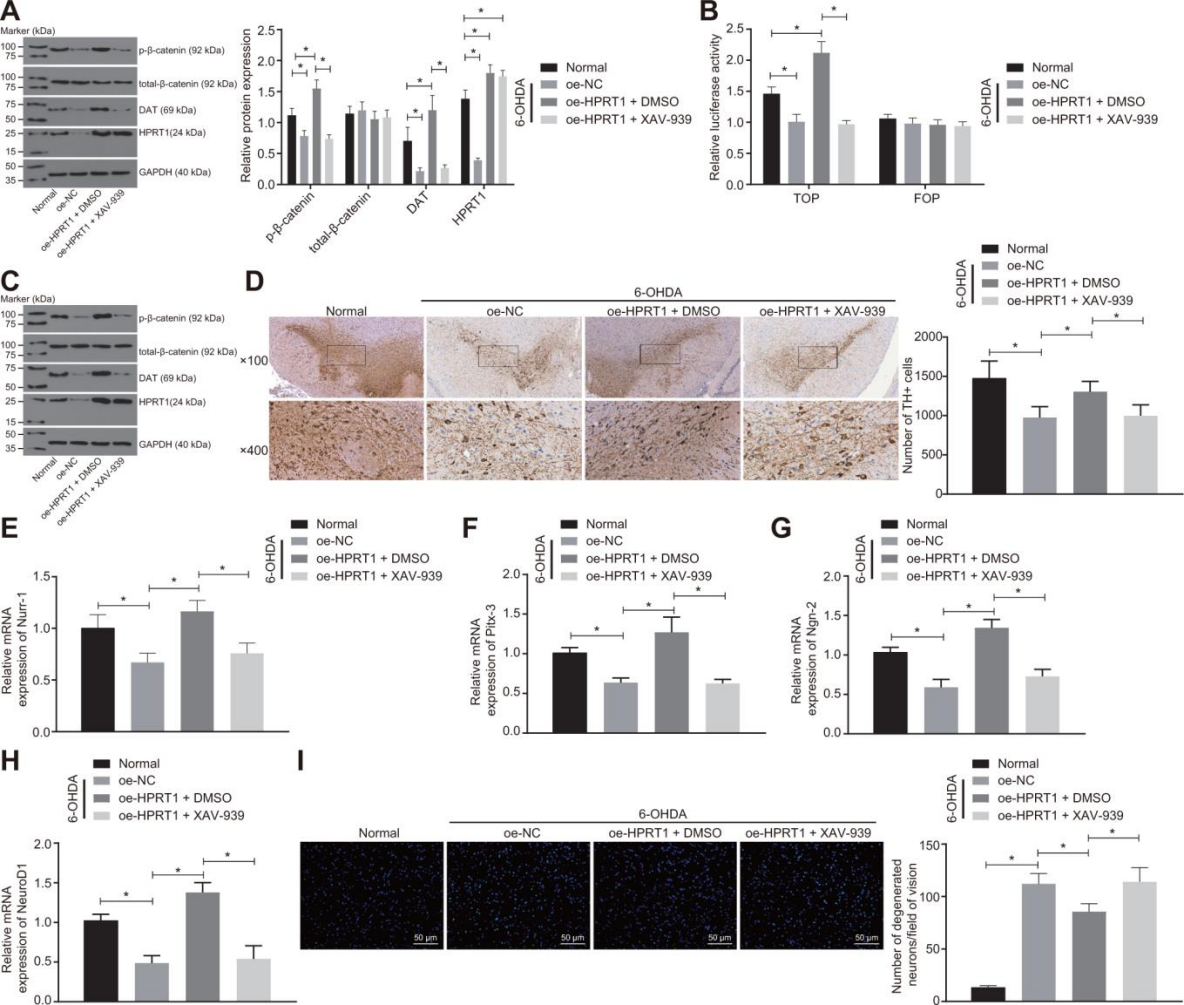
**Overexpressed HPRT1 inhibits dopaminergic neuron loss via the Wnt/β-catenin signaling pathway in 6-OHDA-treated N27 dopaminergic neurons and mice.** N27 dopaminergic neurons and mice were treated with oe-HPRT1 in the presence of the Wnt/β-catenin blocker XAV-939 or DMSO carrier. (**A**) The protein expression of total-β-catenin, DAT and HPRT1 as well as the extent of β-catenin phosphorylation in the N27 dopaminergic neurons detected by western blot assay. (**B**) The activity of the Wnt/β-catenin signaling pathway expressed by TOP/FOP ratio in the N27 dopaminergic neurons. FOP was designated as background value or as negative control due to its stability. (**C**) The protein expression of total-β-catenin, DAT and HPRT1 as well as the extent of β-catenin phosphorylation in the normal and 6-OHDA-lesioned PD mice. (**D**) TH positive neurons in the substantia nigra tissues examined by immunohistochemistry (upper × 100, lower × 400). (**E**–**H**) The mRNA expression of Nurr-1 (**E**), Pitx-3 (**F**), Ngn-2 (**G**) and NeuroD1 (**H**) in the substantia nigra tissues examined by RT-qPCR. (**I**) Fluoro-Jade B-stained apoptotic N27 dopaminergic neurons (scale bar = 50 μm). *p < 0.05. Measurement data are expressed by means ± standard deviation. Comparison between two groups was analyzed by unpaired *t* test, and comparison among multiple groups by one-way analysis of variance. n = 6.

To further confirm it is through the Wnt/β-catenin signaling pathway that HPRT1 exerts effects on PD, the 6-OHDA-treated N27 dopaminergic neurons were transduced with oe-HPRT1 in the presence of XAV-939 or DMSO. The levels of total-β-catenin, HPRT1 and DAT as well as the extent of β-catenin phosphorylation were determined by western blot analysis, which demonstrated that the protein expressions of HPRT1 and DAT, as well as the extent of β-catenin phosphorylation, in 6-OHDA intoxicated N27 dopaminergic neurons was significantly lower than that in normal N27 dopaminergic neurons. After 6-OHDA treatment, the protein expressions of HPRT1 and DAT as well as the extent of β-catenin phosphorylation were elevated by infection of lentiviral oe-HPRT1 and DMSO treatment in N27 dopaminergic neurons, while the transduction of oe-HPRT1 and treatment with the Wnt/β-catenin inhibitor XAV-939 could reverse the promotion of HPRT1 on the extent of β-catenin phosphorylation (Figure 4A). At the same time, the TOP/FOP flash assays also demonstrated that the TOP flash luciferase activity increased in response to the transduction of oe-HPRT1 and DMSO treatment, but did not differ significantly in the presence of oe-HPRT1 and treatment with XAV-939 (Figure 4B). Furthermore, we treated 6-OHDA-induced PD mice with oe-HPRT1 in DMSO carrier or XAV-939. A western blot assay was carried out to assess the protein expression of total-β-catenin, HPRT1 and DAT as well as the extent of β-catenin phosphorylation in these mice; results in mice were consistent with those of the *in vitro* experiment (Figure 4C). Meanwhile, the immunohistochemical analysis showed that, relative to control mice, 6-OHDA-lesioned mice had diminished HPRT1 protein expression, lower extent of β-catenin phosphorylation, fewer TH positive neurons in the substantia nigra (Figure 4D), as well as reduced mRNA expression of Nurr-1 (Figure 4E), Pitx-3 (Figure 4F), Ngn-2 (Figure 4G) and NeuroD1 (Figure 4H). On the other hand, the injection of oe-HPRT1 plus DMSO following 6-OHDA treatment contributed to rescue of TH-positive neurons and mRNA expression of Nurr-1, Pitx-3, Ngn-2 and NeuroD1 in mice compared with oe-NC-injected mice. In contrast, XAV-939 treatment simultaneously with oe-HPRT1 reversed the effects HPRT1 overexpression on the substantia nigra tissues. As assessed by Fluoro-Jade B staining, the apoptosis rate of neurons in the substantia nigra was promoted by 6-OHDA treatment and attenuated by forced overexpression of HPRT1. However, this effect was reversed by treatment with the Wnt/β-catenin signaling pathway inhibitor XAV-939 (Figure 4I). These results suggested that overexpression of HPRT1 inhibits 6-OHDA-induced dopaminergic neuron loss through activating the Wnt/β-catenin signaling pathway.

### H19 regulates HPRT1 expression by sponging miR-301b-3p

**Figure 5:**
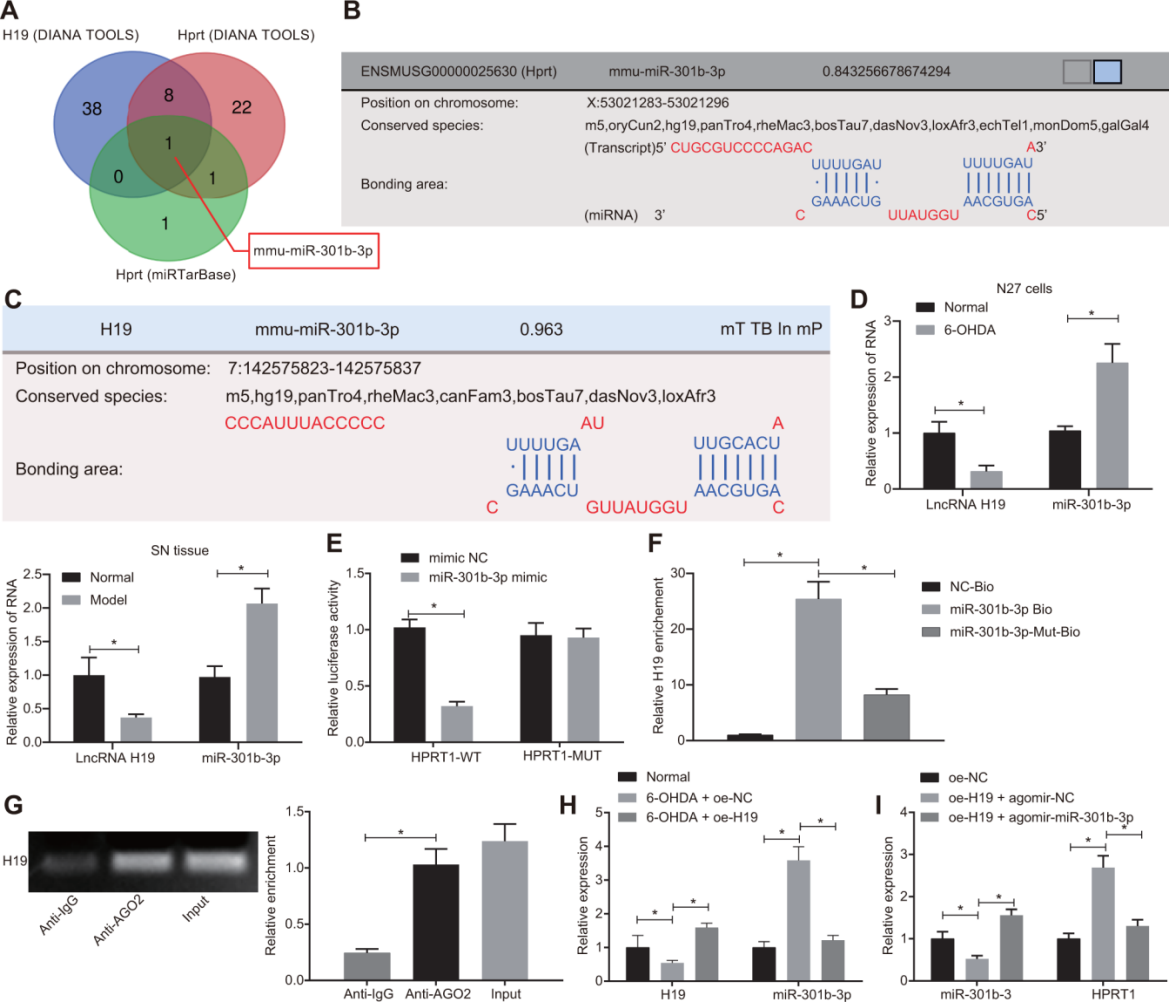
**H19 upregulates HPRT1 expression by inhibiting miR-301b-3p.** (**A**) The predicted miRNAs binding to H19 and HPRT1; (**B**) Putative binding sites between miR-301b-3p and HPRT1 3’UTR; (**C**) Putative binding sites between miR-301b-3p and H19; (**D**) The expression of miR-301b-3p and H19 in N27 dopaminergic neurons and substantia nigra tissues assessed by RT-qPCR. (**E**) Binding of miR-301b-3p and HPRT1 verified by dual-luciferase reporter gene assay. (**F**) N27 dopaminergic neurons transfected with miR-301b-3p-Bio or miR-301b-3p-Mut-Bio or NC-Bio tested by RNA pull-down assay 48 h after transfection. H19 expression was evaluated by RT-qPCR. (**G**) Verification of RIP assay for direct interaction of H19 with Ago2 protein. (**H**) The expression of H19 and miR-301b-3p in the substantia nigra tissues in response to oe-H19 examined by RT-qPCR. (**I**) The miR-301b-3p expression and the mRNA expression of HPRT1 in the substantia nigra tissues in response to oe-H19 in the presence of agomir-miR-301b-3p examined by RT-qPCR. *p < 0.05. Measurement data are by means ± standard deviation. Comparison between two groups was analyzed by unpaired *t* test, and comparison among multiple groups by one-way analysis of variance. n = 6.

LncRNA H19 has been documented to be down-regulated in tissue from PD patients [25]. To investigate the upstream regulatory mechanism of HPRT1, we predicted which miRNAs H19 and HPRT1 should jointly bind through the DIANA TOOLS (http://diana.imis.athena-innovation.gr/DianaTools/index.php?r=site/page&view=software) and miRTarBase (http://mirtarbase.mbc.nctu.edu.tw/php/index.php). Then, the intersection of the two sets was obtained by the online website (http://bioinformatics.psb.ugent.be/webtools/Venn/), which demonstrated that bindings sites likely existed between mmu-miR-301b-3p to H19 and HPRT1 (Figure 5A–5C). Since miR-301b-3p expression is up-regulated in neurologic disorders [26], we selected miR-301b-3p as the research biomarker, to test the hypothesis that H19 might regulate HPRT1 expression by “sponging” miR-301b-3p, thus participating in the 6-OHDA-induced dopaminergic neuron loss. To further elucidate the potential roles of miR-301b-3p and H19 in the 6-OHDA model, we measured the expressions of miR-301b-3p and H19 in N27 dopaminergic neurons and mouse brain by RT-qPCR. As shown in Figure 5D, compared with the normal N27 dopaminergic neurons and mice, the expression of H19 was downregulated and the expression of miR-301b-3p was upregulated in 6-OHDA-treated N27 dopaminergic neurons and in PD-model mice.

In the N27 dopaminergic neurons, we confirmed the binding of miR-301b-3p to HPRT1 on the basis of dual-luciferase reporter gene assay. MiR-301b-3p mimic could reduce the luciferase activity of HPRT1-WT, but failed to influence the luciferase activity of HPRT1-MUT (Figure 5E). An RNA-pull down assay also confirmed that H19 and miR-301b-3p can interact with each other (Figure 5F). Through further verification of RIP assay, we investigated whether H19 could directly interact with Ago2 protein; compared with IgG, the Ago2 recruitment was increased by H19 (Figure 5G). Furthermore, we measured expression of H19 and miR-301b-3p in the substantia nigra tissues by RT-qPCR in 6-OHDA-induced PD mice co-injected with lentiviral oe-NC or oe-H19. The results here indicated that H19 expression was significantly reduced and miR-301b-3p expression was increased in 6-OHDA-induced PD mice injected with oe-NC compared with control mice. On the other hand, oe-H19 injection increased H19 expression and diminished miR-301b-3p expression relative to oe-NC injection in 6-OHDA-induced PD mice (Figure 5H). After the injection of lentiviral oe-H19 + agomir-NC, miR-301b-3p expression was lowered, while HPRT1 mRNA expression was restored relative to that with injection of oe-NC alone. Higher miR-301b-3p expression and lower HPRT1 expression were identified in the substantia nigra tissues of oe-H19 plus agomir-miR-301b-3p-treated PD mice, when compared with that after the injection of oe-H19 + agomir-NC (Figure 5I). Together, these results indicate that H19 upregulated HPRT1 expression by sponging miR-301b-3p.

### Overexpressed H19 activates the Wnt/β-catenin signaling pathway by binding to miR-301b-3p

**Figure 6:**
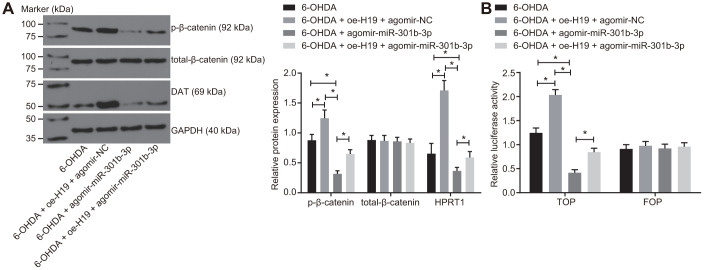
**Overexpressed H19 activates the Wnt/β-catenin signaling pathway by inhibiting miR-301b-3p.** N27 dopaminergic neurons exposed to 6-OHDA were treated with agomir-miR-301b-3p alone or in the presence of oe-H19. (**A**) The protein expression of total-β-catenin and HPRT1 as well as the extent of β-catenin phosphorylation in the N27 dopaminergic neurons detected by western blot assay. (**B**) The activity of the Wnt/β-catenin signaling pathway expressed by TOP/FOP ratio in the N27 dopaminergic neurons. *, *p* < 0.05. Measurement data from three independent experiments are expressed by means ± standard deviation. Comparison between two groups was analyzed by independent sample *t* test.

The N27 dopaminergic neurons were treated with 6-OHDA after transduction of lentiviral oe-H19 and agomir-NC, agomir-miR-301b-3p alone, or oe-H19 together with agomir-miR-301b-3p. The western blot analysis demonstrated that the protein expression of HPRT1 and the extent of β-catenin phosphorylation were elevated in 6-OHDA-exposed N27 dopaminergic neurons when infected with oe-H19 and agomir-NC. The opposite effect was seen in response to infection with oe-H19 and agomir-miR-301b-3p. Infection with agomir-miR-301b-3p also reduced the extent of β-catenin phosphorylation in 6-OHDA-exposed N27 dopaminergic neurons (Figure 6A). According to the TOP/FOP flash assays, the TOP flash luciferase activity increased in response to overexpression of H19 but reduced upon overexpression of miR-301b-3p. Furthermore, the increased TOP flash luciferase activity induced by H19 overexpression was reversed by enhancement of miR-301b-3p (Figure 6B). These results suggested that H19 activates the Wnt/β-catenin signaling pathway by binding to miR-301b-3p.

### H19 inhibits dopaminergic neuron loss by binding to miR-301b-3p

**Figure 7:**
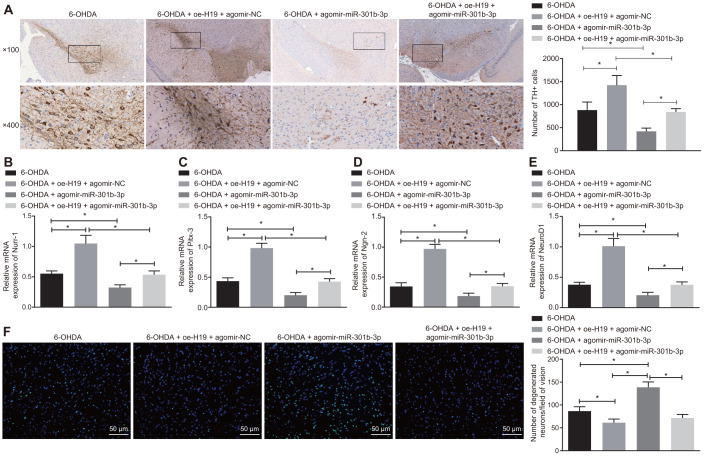
**H19 inhibits dopaminergic neuron loss by inhibiting miR-301b-3p.** 6-OHDA-induced PD model mice were treated with agomir-miR-301b-3p alone or in the presence of oe-H19. (**A**) TH positive neurons in the substantia nigra tissues examined by immunohistochemistry (upper × 100, lower × 400). (**B**–**E**) The mRNA expression of Nurr-1 (**B**), Pitx-3 (**C**), Ngn-2 (**D**) and NeuroD1 (**E**) in the substantia nigra tissues examined by RT-qPCR. (**F**) Fluoro-Jade B-stained apoptotic neurons in the substantia nigra tissues (scale bar = 50 μm). *p < 0.05. Measurement data are by means ± standard deviation. Comparison between two groups was analyzed by independent sample *t* test. n = 6.

Following injection of lentiviral oe-H19 and lentiviral agomir-NC or lentiviral oe-H19 and lentiviral agomir-miR-301b-3p in 6-OHDA-induced PD mice, the number of TH-immunoreactive neurons in the substantia nigra increased after overexpression of H19, but declined with overexpression of miR-301b-3p (Figure 7A). The rescue of TH positive neurons induced by H19 overexpression was reversed by forced up-regulation of miR-301b-3p. Meanwhile, up-regulation of H19 elevated, while up-regulation of miR-301b-3p diminished the mRNA expression of Nurr-1 (Figure 7B), Pitx-3 (Figure 7C), Ngn-2 (Figure 7D) and NeuroD1 (Figure 7E). Additionally, the number of Fluoro-Jade B-stained apoptotic N27 dopaminergic neurons was reduced by overexpression of H19, which was reversed by up-regulation of miR-301b-3p (Figure 7F). In conclusion, H19 inhibited 6-OHDA-induced dopaminergic neuron loss by binding to miR-301b-3p.

## DISCUSSION

**Figure 8:**
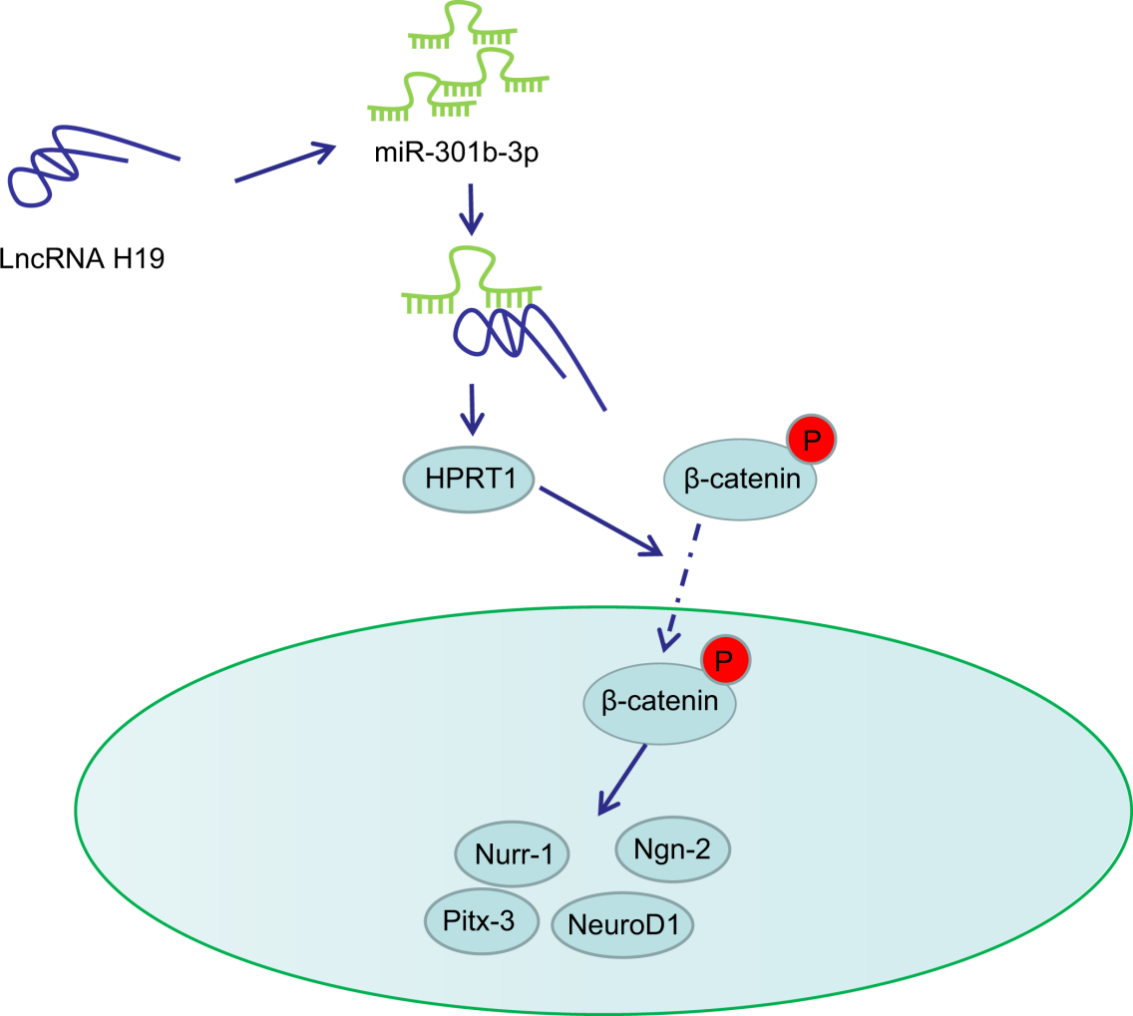
**Schematic representation of H19 in regulating dopaminergic neuron loss in PD.** H19 upregulates the expression of HPRT1 by binding to miR-301b-3p. Overexpression of HPRT1 could activate the Wnt/β-catenin signaling pathway, thus promoting the mRNA expression of Nurr-1, Pitx-3, Ngn-2 and NeuroD1 in the substantia nigra tissues, which ultimately rescues the dopaminergic neuron loss in this 6-OHDA-induced PD mouse model.

PD is a neurodegenerative disorder closely associated with midbrain dopaminergic neuron loss in the substantia nigra pars compacta [27, 28]. The major obstacle for identifying neuroprotective therapies in PD lies in the limited understanding on the dysfunction of molecular events and signaling pathways that stimulate neurodegeneration [29]. In this present study, we provided evidence demonstrating that lncRNA H19 rescues 6-OHDA-induced dopaminergic neuron loss through the activation of the HPRT1-mediated Wnt/β-catenin signaling pathway by inhibiting miR-301b-3p (Figure 8).

The degeneration and reduction of TH positive cells are reported to be the hallmark characteristics of PD [29]. Specifically, TH is a rate limiting enzyme in the dopamine synthesis pathway transforming L-tyrosine to levodopa, and degeneration of the TH-positive nigrostriatal neurons is responsible for the motor symptoms of PD [30, 31]. The 6-OHDA-lesioned PD mice presented significantly down-regulated HPRT1 expression in addition to the expected decrease in TH-positive neurons in the substantia nigra tissues. The most remarkable and widely reported neurophysiological consequence caused by HPRT deficiency for the central nervous system, as in Lesch-Nyhan disease, is impaired development of the dopaminergic nigrostriatal pathway [32]. The association between HPRT deficiency and impaired dopamine neuron growth has been partially characterized by previous studies, which indicated that HPRT deficiency could broadly alter transcription factors related to dopamine neurons [13, 23].

Overexpressed HPRT1, in the presence of lentiviral oe-HPRT1, impeded the dopaminergic neuron loss and neuron apoptosis in 6-OHDA-induced PD mice, accompanied by increased mRNA expression of Nurr-1, Pitx-3, Ngn-2 and NeuroD1. HPRT exerts effects in regulating the expression of transcription factors that are critical for the function and development of dopaminergic neurons, involving various transcription factors including Ngn2 and Mash1, and midbrain dopamine neuron-specific factors (Nurr1 and Pitx3) [33]. Ngn2 has been indicated to provoke the expression of Nurr1, which in turn accelerates the development of immature DA neurons, whereas the mature DA neuronal phenotype could be promoted through interaction with such transcription factors as Pitx3 and Mash1 [34, 35].

Furthermore, we found that the overexpression of HPRT1 impeded dopaminergic neuron loss and neuron apoptosis *via* activating the Wnt/β-catenin signaling pathway in 6-OHDA-induced PD mice. HPRT deficiency enhances changes in the Wnt/β-catenin signaling pathway by mediating cytosolic p-β-catenin and β-catenin translocation into cell nuclei [13]. In this study we observed increased β-catenin phosphorylation, suggesting activation of the Wnt/β-catenin signaling pathway in response to oe-HPRT1 treatment in N27 dopaminergic neurons treated with 6-OHDA. Biological functions of diseased midbrain dopaminergic neurons could be rescued by activating the Wnt/β-catenin signaling pathway in endogenous Wnt-responsive sources [36, 37]. Furthermore, dopamine neurogenesis could be potentiated by manipulating the prenatal dopamine progenitors with inhibitors of β-catenin kinase GSK3β [38]. In the present animal model of PD, the stem cell-derived dopamine neurons were rescued with respect to functional integration, differentiation, and survival following transplantation of fetal neural stem cells by treatment with Wnt5a [39].

Of note, the *in-silico* prediction and verification of RIP and RNA pull-down assays determined bindings sites of both H19 and HPRT1 to miR-301b-3p. Further assays indicated that H19 upregulated HPRT1 expression by binding to miR-301b-3p. In other studies, when the imprinting status of H19 and insulin-like growth factors 2 (IGF2) were manipulated, the development of brain tumors including meningiomas, medulloblastomas, and gliomas could be altered [40]. LncRNA H19 is located within the imprinting gene cluster H19-IGF2 [20]. Moreover, the IGF2-proinsulin precursor (INS)-TH gene cluster found on the telomeric end of chromosome 11 is reported to be a region encoding various proteins significant for the homeostasis of dopamine neurons; this same gene cluster is associated with the risk of PD [41]. Interestingly, miR-301b was proved to be significantly increased in the substantia nigra pars compacta of PD model mice, and miR-301b decreased α-synuclein mRNA levels [21]. Therefore, when the miR-301b-3p was sponged by H19, the dopaminergic neuron loss could be curtailed, ultimately slowing down the nigrostriatal degeneration. Furthermore, the up-regulated HPRT1 expression due to H19 sponging miR-301b-3p exerted neuroprotection against dopaminergic neuron loss in the 6-OHDA-induced PD model.

The evidence provided by our study highlights the involvement of lncRNA H19 in the up-regulation of HPRT1 as well as the activation of the Wnt/β-catenin signaling pathway by binding to miR-301b-3p, which can rescue dopaminergic neuron loss in the 6-OHDA-induced PD model. This may therefore guide researchers to productive novel fields of research aiming to understand the neurological defects associated with dopaminergic neuron loss in PD and other neurodegenerative diseases. Further studies are needed to elucidate the details of the H19/miR-301b-3p/HPRT1/Wnt/β-catenin axis underlying our observations.

## MATERIALS AND METHODS

### Microarray-based analysis

The gene expression omnibus (GEO) database (https://www.ncbi.nlm.nih.gov/geo/) was employed to obtain the expression profiles GSE20141 [42] and GSE20168 [43] in relation to PD. The GSE20141 consisted of 8 normal samples and 10 PD samples, whereas the GSE20168 consisted of 15 normal and 14 PD samples, with the normal samples as the control material. The R. package “Limma” was used for differential expression analysis to screen out differentially expressed genes, with |log FoldChange| > 1 and *p* value < 0.05 as threshold values.

### PD models established in mice

Totally, 54 male C57BL/6J mice (aged 10-12 weeks, weighing 23-28 g) were housed in standard temperature and humidity, with circadian rhythm and free access to food and water. Under anesthesia with 1.5% pentobarbital sodium, the mice were fixed in the stereotactic frame. Next, 2 μL 6-hydroxydopamine (6-OHDA; H4381, 3 μg/μl, Sigma Aldrich, St. Louis, MO, USA) dissolved in sterile normal saline with 0.02% ascorbic acid was stereotaxically infused into the substantia nigra (from bregma: AP, −3.0 mm; ML, −1.2 mm; DV, −4.7 mm) [44]. The infusion was applied at a flow rate of 0.5 μl/min. The control group was similarly treated, but infused with sterile normal saline containing 0.02% ascorbic acid into the substantia nigra [22].

### Animal treatment

Following the modeling of the 6-OHDA-induced PD in mice, the mouse models were injected with lentiviruses harboring overexpressed HPRT1 (oe-HPRT1 group), overexpressed H19 (oe-H19), agomir-miR-301b-3p and overexpression vector negative control (oe-NC), agomir negative control (agomir-NC). XAV-939, an inhibitor of the Wnt/β-catenin signaling pathway (10 μM, S1180, Selleck, Shanghai, China) in dimethyl sulfoxide (DMSO) served as control [45]. The lentiviruses of oe-NC, oe-HPRT1, oe-H19, agomir-NC and agomir-miR-301b-3p were purchased from Shanghai GenePharma Co., Ltd. (Shanghai, China). The injections of lentiviruses and 6-OHDA were performed simultaneously. The mice were anesthetized with pentobarbital sodium intraperitoneally and fixed in the stereotactic equipment. A total of 2 μL lentivirus (2.1 × 10^7^ TU/mL) was injected into the substantia nigra at an injection flow rate of 0.5 μL/min.

### Sample collection

At 14 day after administration of 6-OHDA, the mice were anesthetized with pentobarbital sodium. Following treatment of 0.9% sterile normal saline, the brain was fixed by perfusion with 4% paraformaldehyde, followed by post-fixation in PFA for 24 h and then cryopreservation in 30% sucrose. The brains were coronally sliced at a thickness of 20 μm after being embedded. The brain slices were preserved at -20°C for immunofluorescence staining. The 5 mice in each group were euthanized to extract brain tissues, from which the substantia nigra was immediately collected on the ice.

### RNA extraction and quantitation

The Trizol method (16096020, Thermo Fisher Scientific, New York, NY, USA) was employed to extract the total RNA of substantia nigra and dopaminergic neurons. A total of 5 μg total RNA was reversely transcribed into cDNA according to the instructions of a cDNA kit (K1622, Fermentas, Ontario, Canada). With cDNA as template, TaqMan MicroRNA Assay and TaqMan^®^ Universal PCR Master Mix were used for quantitative reverse transcription polymerase chain reaction (RT-qPCR). The miRNA expression was determined by RT-qPCR using U6 mRNA levels for normalization.

The RT-qPCR was performed according to the instructions of TaqMan Gene Expression Assays protocol (Applied Biosystems, Foster City, CA, USA), with the glyceraldehyde-3-phosphate dehydrogenase (GAPDH) gene as the internal reference. Each RT-qPCR was repeated in three wells. The primers are displayed in Table 1. The relative expression of each gene was determined by the 2^-ΔΔCT^ method. ΔΔCt = ΔCt _the experimental group_ - ΔCt _the control group_ and ΔCt = Ct _targeted gene_ – Ct _internal reference_. The experiment was independently conducted three times.

**Table 1:** Primer sequences for quantitative reverse transcription polymerase chain reaction.

**Gene**	**Primer sequence (5’-3’)**
HPRT1	F: TCCTCCTCAGACCGCTTTT
	R: CCTGGTTCATCATCGCTAATC
Nurr-1	F: TCAGAGCCCACGTCGATT
	R: TAGTCAGGGTTTGCCTGGAA
Pitx-3	F: TTTCGCAACGGGTTTGCCGC
	R: AAGGTCGCCTCTAGCTCCTGTAG
Ngn-2	F: AGGACGGCTCTCTGAAGAA
	R: TTGACCGAGTTGAAGGCGAA
NeuroD1	F: ATGAACGCAGAGGAGGACTCACTG
	R: TTGGTGGTGGGTTGGGATAAGC
H19	F: GCGAGGTAGAGCGAGTAGCTG
	R: CCTCTGCTGGAGACCCTAGT
miR-301b-3p	F: ATACTCGAGATCCTAGTTTGATACTCCCAGTCTT
	R: TGTTCTAGACATATTTACTTTTATATTTCCATAC
U6	F: CTCGCTTCGGCAGCACA
	R: AACGCTTCACGAATTTGCGT
GAPDH	F: CGTCCCGTAGACAAAATGGT
	R: TTGATGGCAACAATCTCCAC

Note: F, forward; R, reverse; HPRT1, hypoxanthine phosphoribosyltransferase 1; Pitx-3, paired-like homeodomain transcription factor 3; Ngn-2, neurogenin 2; NeuroD1, neurogenic differentiation 1; miR-301b-3p, microRNA-301b-3p; GAPDH, glyceraldehyde-3-phosphate dehydrogenase.

### Western blot assay

The total protein of the *substantia nigra* and dopaminergic neurons were extracted by radio-immunoprecipitation assay (RIPA) lysis buffer (R0010, Solarbio, Shanghai, China) containing phenylmethylsulfonyl fluoride (PMSF). The total protein was incubated and centrifuged to collect the supernatant, the total protein concentration in which was determined by bicinchoninic acid kit. A total of 50 μg protein dissolved in 2 × sodium dodecyl sulfate (SDS) uploading buffer was boiled at 100°C for 5 min. Then the cooled protein sample was loaded onto 10% SDS-polyacrylamide gel electrophoresis. After separation, the proteins were transferred onto a polyvinylidene fluoride (PVDF) membrane, which was blocked 5% skim milk for 1 h at room temperature. Next, the PVDF membrane was incubated with diluted GAPDH (ab9485, 1:2500, Abcam, Cambridge, UK), tyrosine hydroxylase (TH; MAB318, 1:2000, Millipore, Bedford, MA, USA), HPRT1 (ab10479, 1:1000, Abcam), β-catenin (ab32572, 1:5000, Abcam), phosphorylated (p-)β-catenin (ab27798, 1:500, Abcam) and dopamine transporter (DAT; ab111468, 1:1000, Abcam) at 4°C overnight. The membranes were then incubated with horseradish peroxidase-labeled secondary antibody for 1 h. Proteins were visualized with the enhanced chemiluminescence kit (BB-3501, Amersham, Little Chalfont, Buckinghamshire, UK). The assay was carried out using an eluent (P0025B, Beyotime Biotechnology Co., Ltd., Shanghai, China) for extraction of of the developed bands. Here, the bands were placed in an antibody incubation box containing 3 mL eluent in a shaking table for 5 min. After being shaken with 5 mL Tris-buffered saline with Tween 20, the bands were blocked with 5% milk for 1 h and then subjected to the primary antibody incubation. The images were captured in a Bio-Rad Gel Doc EZ Imager (Bio-Rad, Hercules, CA, USA) and quantified with Quantity One v4.6.2 software. The relative protein levels were represented by gray value of protein bands / gray value of protein bands of GAPDH.

### Immunohistochemistry

At day 14 after administration of 6-OHDA, the mice were anesthetized with pentobarbital sodium. After perfusion with 0.9% normal saline, the brains were post-fixed in 4% paraformaldehyde for 24 h and then cryopreserved in 30% sucrose. The brain tissues of substantia nigra were sliced at a thickness of 10 μm. After the washing step, the slices were permeabilized in TBS containing 0.2% TBST at room temperature for 30 min. Following blocking in TBST with 5% bovine serum albumin for 2 h, the slices were incubated with the primary antibody against TH (MAB318, 1:2000, Millipore, Billerica, MA, USA) at 4°C overnight. The quantitation of cells was performed by a person blind to the experimental groups and conditions. Six mice in each group were analyzed to determine.

In the whole substantia nigra of each mouse, the number of TH^+^ neurons was calculated from every 6 transverse sections. Each section was observed under lower magnification and outlined. Then the number of TH^+^ cells was counted at higher magnification. Image-Pro+ 6.0 (Media Cybernetics, Bethesda, MD, USA) was used to measure the cross-sectional area of the substantia nigra, and the number of TH^+^ cells per square millimeter was calculated for each section [46].

### Fluoro-Jade B staining

Fluoro-Jade B staining was performed using a Fluoro-Jade B staining kit (AAT Bioquest, Sunnyvale, CA, USA) as described in the manufacturer’s instructions. In brief, the fixed brains were sliced into 25-μm sections and mounted. The sections were immersed in 1% NaOH with 80% ethanol for 5 min, followed by immersion in 70% ethanol for 2 min, and in ddH_2_O for 2 min. After being rinsed with distilled water, the sections were reacted with 0.06% potassium permanganate solution for 10 min followed by another 2-min wash in ddH_2_O. The sections were then stained with 0.0004% Fluoro-Jade B staining solution for 20 min and permeabilized with 0.1% Triton X-100. The apoptotic neurons were examined using an epifluorescent microscope at 490/525 nm. For each animal, three or four fields of view in the substantia nigra were selected randomly, and two fields were observed for quantification.

### Cell treatment and grouping

N27 dopaminergic neurons (SCC048, EMD Millipore, Merck Life Science (Shanghai) Co., Ltd., Shanghai, China) were incubated with Roswell Park Memorial Institute 1640 medium. The medium was renewed every other day. After 24 h, the cells were fed with fresh medium and treated with 100 mM 6-OHDA [47] dissolved in distilled water and/or lentivirus overexpressing negative control (oe-NC group), HPRT1 (oe-HPRT1 group), H19 (oe-H19 group), H19 and agomir negative control (oe-H19 + agomir-NC group), H19, and miR-301b-3p agomir (oe-H19 + agomir-miR-301b-3p group).

### TOP/FOP flash reporter assay

One day before transduction, N27 dopaminergic neurons (SCC048, EMD Millipore) were seeded in the 24-well plate at a density of 5 × 10^4^ neurons/well and incubated for 24 h. TOP flash (or FOP flash) lentiviruses and the internal control *Renilla* luciferase were further incubated for 24 h. Then, the neurons were separately treated into four different groups for 24 h: (1) oe-NC, (2) oe-HPRT1, (3) oe-H19 + agomir-NC, and (4) oe-H19 + agomir-miR-301b-3p. The neurons were inoculated in the white light-proof 96-well plate. According to the instructions of a Dual-Glo Luciferase Assay System kit (E2920, Promega, Madison, WI, USA), the activity of the Firefly luciferase/*Renilla* luciferase was identified and quantitated. The activity of the Wnt/β-catenin signaling pathway was expressed by TOP/FOP ratio. FOP was designated as background value or as NC due to its stability

### Dual-luciferase reporter gene assay

The synthesized HPRT1 3’untranslated region gene fragments were cloned to the pMIR-reporter (Huayueyang Biotechnology Co., Ltd., Beijing, China) between SpeI and Hind III. The mutant sites of complementary sequence of the seed sequence were designed on the wild type HPRT1 (HPRT1-WT). Through restriction endonuclease, the target fragments were inserted into the pMIR-reporter plasmid by using T4 DNA ligase. The luciferase reporter plasmids HPRT1-WT and mutant HPRT1 (HPRT1-Mut) were separately co-transfected with miR-301b-3p to the HEK-293T cells (CRL-1415, Shanghai Xin Yu Biotech Co., Ltd., Shanghai, China). At 48 h after transfection, the lysate of cells was detected by a luciferase detection kit (RG005, Beyotime Institute of Biotechnology, Shanghai, China), with luciferase activity identified by the Glomax20/20 luminometer (Promega Corporation, Madison, WI, USA). The relationship between miR-301b-3p and HPRT1 was also assessed by the same method. The experiment was independently conducted three times.

### RNA immunoprecipitation (RIP)

The N27 dopaminergic neurons (SCC048, EMD Millipore) were lysed by the lysis buffer containing 25 mM Tris-HCl (pH = 7.4), 150 mM NaCl, 0.5% NP-40, 2 mM ethylenediaminetetraacetic acid, 1 mM NaF and 0.5 mM dithiothreitol supplemented with RNasin (Takara Biotechnology, Dalian, Liaoning, China) and proteinase inhibitor cocktail (B14001a, Roche Molecular Systems, Inc., Alameda, CA, USA). The lysate was centrifuged to collect the supernatant, which was mixed with Ago2 magnetic beads (130-061-101, Univ-bio Inc., Shanghai, China). The control group was added with anti-IgG magnetic beads. After a 4-h incubation at 4°C, the magnetic beads were washed three times with the buffer containing 50 mM Tris-HCl, 300 mM NaCl (pH = 7.4), 1 mM MgCl_2_, 0.1% NP-40. RNA was extracted from magnetic beads by the Trizol method, and H19 and miR-301b-3p expression was examined by RT-qPCR.

### RNA pulldown assay

Biotin-labeled RNAs were transcribed with the Biotin RNA Labeling Mix (Roche Diagnostics, Indianapolis, IN, USA) and T7 RNA polymerase (Roche Diagnostics, Indianapolis, IN, USA), treated with RNase-free DNase I (Roche Diagnostics, Indianapolis, IN, USA), and purified with a RNeasy Mini Kit (Qiagen, Valencia, CA, USA). Subsequently, 1 mg whole-cell lysate from N27 dopaminergic neurons was incubated for 1 h with 3 μg purified biotinylated transcripts at 25°C. Complexes were extracted with streptavidin agarose beads (Invitrogen Inc., Carlsbad, CA, USA). The retrieved protein was detected using the conventional western blot assay.

### Statistical analysis

SPSS 21.0 software (IBM Corp. Armonk, NY, USA) was used for statistical analysis. The measurement data were displayed as mean ± standard deviation. Comparison between two groups was analyzed by independent sample *t* test, and comparison among multiple groups by one-way analysis of variance (ANOVA). *p* < 0.05 was an indication of significant difference.

### Ethics statement

The animal experimental processes were approved by the Ethnic Committee of Shengjing Hospital of China Medical University (approval number: 201802003) and conducted in strict accordance to the Guide for the Care and Use of Laboratory Animals published by the National Institutes of Health.
